# Using Proxy Records to Document Gulf of Mexico Tropical Cyclones from 1820-1915

**DOI:** 10.1371/journal.pone.0167482

**Published:** 2016-11-29

**Authors:** Jordan V. Pino, Robert V. Rohli, Kristine L. DeLong, Grant L. Harley, Jill C. Trepanier

**Affiliations:** 1 Department of Geography and Anthropology, Louisiana State University, Baton Rouge, Louisiana, United States of America; 2 Department of Geography and Geology, University of Southern Mississippi, Hattiesburg, Mississippi, United States of America; Universidade de Vigo, SPAIN

## Abstract

Observations of pre-1950 tropical cyclones are sparse due to observational limitations; therefore, the hurricane database HURDAT2 (1851–present) maintained by the National Oceanic and Atmospheric Administration may be incomplete. Here we provide additional documentation for HURDAT2 from historical United States Army fort records (1820–1915) and other archived documents for 28 landfalling tropical cyclones, 20 of which are included in HURDAT2, along the northern Gulf of Mexico coast. One event that occurred in May 1863 is not currently documented in the HURDAT2 database but has been noted in other studies. We identify seven tropical cyclones that occurred before 1851, three of which are potential tropical cyclones. We corroborate the pre-HURDAT2 storms with a tree-ring reconstruction of hurricane impacts from the Florida Keys (1707–2009). Using this information, we suggest landfall locations for the July 1822 hurricane just west of Mobile, Alabama and 1831 hurricane near Last Island, Louisiana on 18 August. Furthermore, we model the probable track of the August 1831 hurricane using the weighted average distance grid method that incorporates historical tropical cyclone tracks to supplement report locations.

## Introduction

The HURricane DATabase (HURDAT) of the National Oceanic and Atmospheric Administration (NOAA) National Hurricane Center contains information on Atlantic basin tropical cyclones since 1851 [1]. HURDAT is considered a comprehensive database, especially during the late 20th century; however, it is not without inaccuracies [2]. The temporally increasing trend in tropical cyclone frequency since the late 1800s is often mistakenly assumed to be the result of anthropogenic climate change, but it is suggested that this trend is more likely the result of incomplete tropical cyclone reporting due to the limited observational technology of the pre-flight (1944-present) and pre-satellite (1966-present) eras [3]. Moreover, ships that observed tropical cyclones may have been lost at sea during storms along with their captain’s logs and weather reports that contain cyclone information. Others agree that tropical cyclone frequencies are likely underestimated due to missing storms, and that the lack of traffic outside shipping lanes causes many non-landfalling tropical cyclones to be missed [2]. Corroborating those findings, studies note that HURDAT in the pre-satellite era is likely to have missed some category 4 and 5 tropical cyclones due to limited surface observations [4]. In analyzing the “best track” data via a survey of hurricane specialists charged with updating the database, a new format (HURDAT2; version 2) is designed to reduce uncertainties in wind radii and intensities (http://www.aoml.noaa.gov/hrd/hurdat/Data_Storm.html) [5].

Historical documents provide a valuable resource for tropical cyclone research because they allow a glimpse into the near past, a time interval that is of interest to many. One of the first noteworthy attempts at using historical documents to aid in tropical cyclone detection is Poey’s [6] listing of 400 tropical cyclones from 1493–1855 in the West Indies and North Atlantic Ocean. A quality control study of those 400 tropical cyclones is conducted via historical newspaper accounts, weather diaries, and ship logbooks [7]. Historical records also form the basis of Fraser’s [8] narrative of tropical cyclones since 1686 that struck Georgia and South Carolina in the United States (US). Various historical documents reveal tropical cyclones along the US Gulf of Mexico coast from 1851–2007 [9] and elsewhere in the Atlantic from 1851–1898 [10]. Spanish historical documents in the General Archive of the Indies in Seville identify other previously unreported hurricanes in the Caribbean from 1600–1800 [11]. Historical documents can also provide additional details on particular known storms [12–14]. Beyond documenting storm occurrences, historical documents can be used to generate probable tracks of past Atlantic hurricanes from currently archived storm tracks [15]. These studies demonstrate the utility in using historical documents to identify past tropical cyclones before modern technological advances.

Given the temporal limitations of the HURDAT2 database [5] and the utility of historical documents, this study identifies and reviews US Army Fort historical documents (1820–1915) and other archived documents for the northern Gulf of Mexico (Texas, Louisiana, Mississippi, Alabama, and west coast of Florida) for additional information on landfalling tropical cyclones. We also attempt to discover currently undocumented tropical cyclones and create probable track reconstructions. Along with historical documents, we incorporate a tree-ring reconstruction from the Florida Keys (1707–2009) to compliment documentary evidence. These results will benefit tropical cyclone researchers, historians, and environmental risk assessors, and may be included in updates to HURDAT2 and other tropical cyclone databases.

## Materials and Methods

The NOAA Climate Database Modernization Program’s online archive EV2 (https://www.ncdc.noaa.gov/EdadsV2/) is used to detect tropical cyclones along the US Gulf Coast. That database includes a large array of historical documents saved as scanned images, including Army fort meteorological records, storm reports, plantation diaries, and ship logbooks. The Army fort records date back to 1814, when the first nationwide weather system of the US Federal Government was established when Army hospital, post, and regimental surgeons were directed to keep records of the weather. They made several daily observations of temperature, wind direction and speed, cloudiness, and precipitation recorded on a standardized “Meteorological Register.” These Army weather observers have experience using thermometers and other scientific instruments as well as advanced education and training on making meteorological observations. Army fort records from the northern Gulf Coast prior to 1915 are supplemented by other archived documents when an event was identified. The keywords “hurricane,” “heavy rain,” “heavy wind,” “flooding,” “flood damage,” “deaths,” “gale,” “calmness” (during the eye), and “surge” are used to ascertain whether a weather event was likely tropical in nature. Because these keywords could also be associated with non-tropical cyclone weather events, care is taken to use common tropical cyclone characteristics such as time of year, wind direction, and duration to confirm its tropical nature. Given that the words “hurricane” or “tropical cyclone” may not appear in many of the documents, these keywords are necessary substitutions. Any document containing any of the keywords listed above is then investigated further to determine the storm type. Possible tropical cyclones are corroborated with other sources (i.e., publications, books, newspapers). For these storms, no attempt is made to distinguish between tropical storm and hurricane, or to infer the Saffir-Simpson category due to the limited information available.

**Fig 1:**
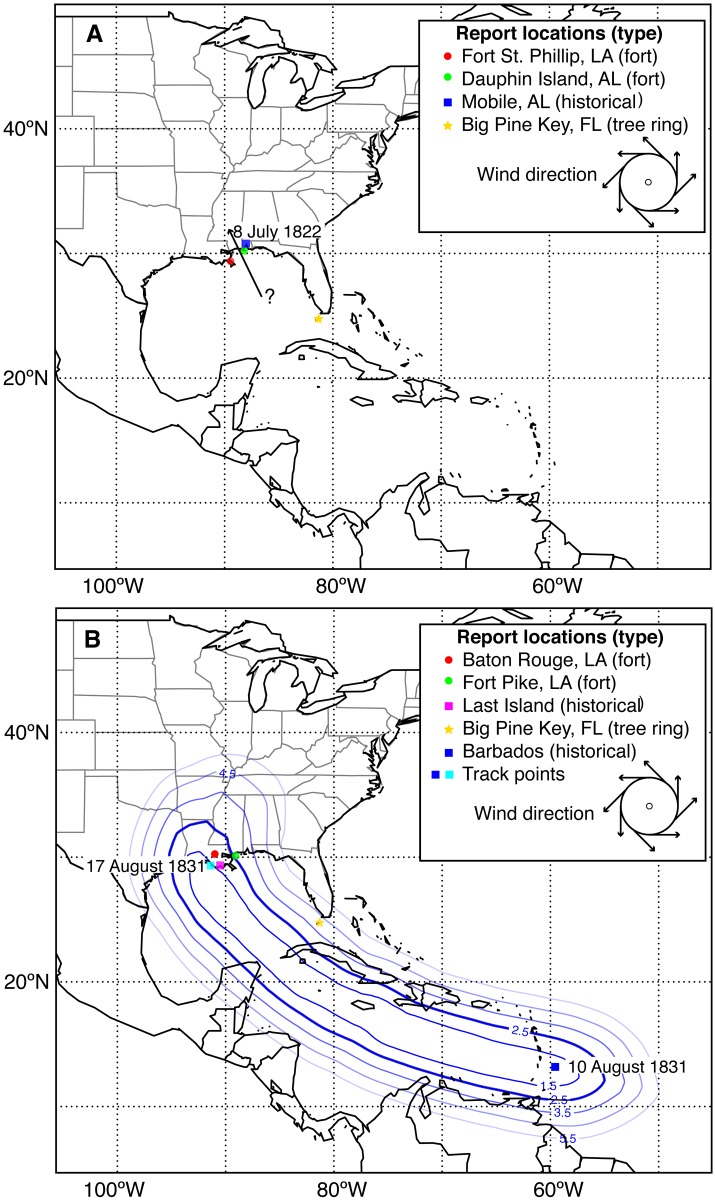
Landfall reconstruction for (a) the hurricane of 8 July 1822 and (b) storm track reconstruction for the hurricane of 10–17 August 1831. (b) Observations of this hurricane include fort and historical documents (this study and Chenoweth [7]) and a tree-ring reconstruction [16]. Storm track is constructed using a weighted average of the distance grids of all track analogs. Bold line encompasses a weighted average distance of ≤ 2.5° of latitude. Contour lines are drawn at 1° increments out to 6.5°. Wind direction is noted via the wind barbs on the hurricane graphic.

Tropical cyclone events identified with fort and other archived documents are compared to a tree-ring based hurricane reconstruction (1707–2009) for Big Pine Key in the Florida Keys (24°42′09″N, 81°23′22″W; see Fig 1 for location) based on 38 slash pine (*Pinus elliotti* var. *densa*) trees [16]. Trees in this location are sensitive to high winds and storm surge induced saltwater intrusion associated with tropical cyclone passage with 91% of passing tropical cyclones between 1851–2009 recorded as a growth suppression in the rings [16]. However, the annual resolution of the tree-ring record cannot assign a month or day for a particular event. We include the tree-ring reconstruction because of the proximity to Fort Jefferson in the Dry Tortugas, Florida (1861–1873), and to Fort Taylor in Key West, Florida (1829–1891), where historical Army documents for the years indicated are available for comparison. Additionally, this location can provide further information on storm tracks, as many tropical cyclones pass through the Florida Straits before entering the Gulf of Mexico.

We use information from historical documents to develop an average storm track using the method described by Scheitlin et al. [15] for the storm of 1831. The average track is created using a weighted average of the distance grids of all track analogs (1851–2014; S2 Table) that passed through our points of documented tropical cyclone occurrence.

## Results

Examination of Army fort records and other EV2 archives (S1 Table) reveals 28 tropical cyclones (Table 1). Twenty of the tropical occurrences found in this study (Table 1) are included in HURDAT2 [5] and/or are documented by other studies [7, 9, 10, 12, 14, 17], but we provide additional information. We also identify seven tropical cyclones that occurred before 1851, three of which are potential new documentations. One storm, in May 1863, is revealed in the historical records, but even though it occurred in the HURDAT2 era (i.e., 1851 –present), it is absent from the HURDAT2 database. A fort record from Key West, Florida indicates “heavy squalls of wind and rain” on 28 May 1863. While occurring very early in the “season,” it is possible this storm was tropical in nature. This event is well documented in a previous study, with a probable track given [14].

**Table 1:** Tropical cyclones identified.

Tropical cyclones concurrent with HURDAT2				
Year	Day Month	Likely Status (landfall)	Location	Quotes from documents
1915	28–31 September	Hurricane	Louisiana	Storm caused over 12 inches of rain in the Florida parishes of Louisiana; 8 inches in New Orleans
1909	19–22 September	Hurricane	Louisiana	Damage from wind and tide was reported from Pensacola, FL to Galveston, TX; 350 persons killed
1908	16–20 September	Tropical Storm	Louisiana	Heavy rain and wind along Texas/Louisiana coast; 62 mph near Galveston. Heavy rain in Cameron Parish; intense burst of rain near New Orleans
1902	26–30 June	Hurricane	Texas	Heavy rain totals; 8 inches in some areas
1898	2–4 October	Tropical Storm	Florida	Heavy rain totals recorded in FL, GA, and SC
1896	6–8 July	Hurricane	Florida	Map of storm track; heavy rain totals
1894	24–26 September	Hurricane	Florida	Heavy rain; over 11 inches of rain at some stations
1890	26 August	Tropical Storm	Louisiana	Strong wind from the northeast
1888	8–12 September	Tropical Storm	Florida	Storm entered Florida near Cedar Key with maximum winds near 60 mph; lowest pressure was 29.50 inches
1888	19–20 August	Hurricane	Louisiana	Remarkable storm
1887	17–18 October	Hurricane	Louisiana	Heavy wind
1880	13 August	Hurricane	Texas	Gale
1879	1 September	Hurricane	Louisiana	The great storm of the 1st….
1877	17–19 September	Hurricane	Louisiana	Heavy storms; wind and rain
1875	15 September	Hurricane	Texas	Heavy rain with wind from the north and northeast
1870	19–20 October	Hurricane	Fort Jefferson, Tortugas, Florida	Trees and fences prostrated, buildings unroofed, & debris flying in the every direction, making it dangerous to be out. At 8:15 A.M. the wind died completely out in 3 minutes, so close as to be uncomfortable, Suddenly at 9:40 A.M. it 1st in from the opposite direction, and in 20 minutes increased to a Hurricane
1865	13 September	Hurricane	Texas	Wind 2–4 a.m. all night
1863	28 May	Hurricane	Florida	Heavy squalls of wind and rain
1859	19 September	Hurricane	Louisiana	Heavy rain
1856	10–12 August	Hurricane	Louisiana	Illegible; rain mentioned
1852	24–25 August	Hurricane	Alabama	Squally
1852	24–25 August	Hurricane	Mississippi	Illegible; wind mentioned
**Pre-HURDAT2 tropical cyclones**				
1837	6–7 October	Hurricane	Louisiana	High winds, rainy day; high winds
1831	16–18 August	Hurricane	Louisiana	Heavy rain and high winds throughout; winds from southeast on 17^th^, then southwest on 18^th^
1831	16–18 August	Hurricane	Louisiana	Heavy winds; river overflowing; storm continues with great violence; winds from southeast
1831	27–29 August	Tropical Storm	Louisiana	Heavy rain; high winds from the northeast throughout; river overflowing
1828	11–12 August	Potential	Louisiana	Rain and wind; mentioned 2 days in a row
1825	21 September	Potential	Louisiana	Windy and rainy
1822	8–9 July	Hurricane	Florida	Storm
1822	7–9 July	Hurricane	Louisiana	Heavy winds which increased to great violence on the 8^th^; winds from the northeast
1822	7–9 July	Hurricane	Mississippi	Rain becoming a gale; winds from the east
1820	24–25 August	Potential	Mississippi	Rain mentioned 2 days straight

Among the storms included in HURDAT2, the earliest is an 1852 landfall along the Mississippi-Alabama coast. The latest hurricane (1915) is noted in a Louisiana storm report as having dumped “over 12 inches of rain in the Florida parishes of Louisiana; 8 inches in New Orleans.” A fort record from Baton Rouge, Louisiana (30°27′29″N, 91°08′25″W), contains a description of intense rainfall related to the August 1856 hurricane documented by Sallenger [12]; however, much of that record was illegible. We do not find any other sources that mention the 1856 hurricane, possibly because its intense nature obliterated documents that reported it.

We identify seven pre-HURDAT2 (pre-1851) tropical cyclones, including two records for the 1822 hurricane, three records for two tropical cyclones in 1831, and one in 1837 (Table 1). The hurricane of 1822 made landfall between 7–9 July as reported in a record from Fort St. Philip (29°21′47″N, 89°27′57″W) in Plaquemines Parish, Louisiana, about 64 km upriver from the mouth of the Mississippi. One of the storms of 1831 is described in a fort record from Baton Rouge, Louisiana (30°27′29″N, 91°08′25″W), where rivers are noted to be overflowing. The other 1831 tropical cyclone made landfall in Louisiana with overflowing rivers and high winds noted in fort records. A fourth likely tropical system made landfall in Louisiana in October 1837, according to a record from Fort Jesup (31°36′41″N, 93°24′03″W) located 35 km west of Natchitoches, Louisiana, that mentioned high winds and rain. No description of this storm is found in documents from the southern part of the state.

Three possible tropical cyclones were identified in the Army Fort registers (Table 1). The first appears in the fort record from Bay St. Louis, Mississippi for 24–25 August 1820 (30°18′31″N, 89°19′48″W) that describes rain for two consecutive days. The second occurs on 21 September 1825 in the Fort Petite Coquille, Louisiana record that notes severe rain and wind. Finally, a record from Fort Pike, Louisiana, from 11–12 August 1828 mentions rain and wind for two consecutive days. No other fort records reviewed in this area mention these events; however, the early 1820s is after the US took possession of the Louisiana territory in 1803 and after the War of 1812–1815 when US Army forts started to be continuously garrisoned in the state.

Storm track reconstructions for two tropical cyclones are made based on evidence from documents reviewed along with the tree-ring reconstruction. The hurricane of July 1822 was previously described as making landfall near Mobile, Alabama, based on newspaper reports and logbooks [7]. We found three fort records to corroborate this landfall location, one from Fort St. Philip, Louisiana (29°21′47″N, 89°27′57″W), the second from Dauphin Island, Alabama (30°15′15″N, 88°06′44″W), and the third from Camp Hope in west Florida (30°23'52"N, 87°14'27"W). The record from Fort St. Philip notes “heavy winds which increased to great violence” on 8 July with wind direction from the northeast. The record from Dauphin Island mentions that a “gale had begun on 8 July with winds from the east.” The third record from Camp Hope notes a “Storm” on 8–9 July, with winds from the southeast and rain. Shipwreck records indicate the sloop Lady Washington ran aground on Ship Island (30°12'37"N, 88°57'45"W) on 7 July during this hurricane [18]. These findings support Chenoweth’s landfall location near Mobile, with easterly winds north of the eye as the storm makes landfall from the south and northeasterly winds on the west side of the eye (Fig 1a) [7]. These two forts, located 168 km apart, suggest a large wind field for this hurricane. The tree-ring reconstruction from Big Pine Key, Florida, indicates that 92% and 77% of trees are suppressed during 1822 and 1823, respectively [16]; however, that reconstruction cannot identify the month of occurrence and thus is used to compliment the documentary records. This finding suggests that a hurricane passed near the Florida Keys in 1822 but fort records are not available from the Keys to confirm this storm’s date. Given the evidence gathered for the hurricane of 1822, the track reconstruction is limited to a landfall location near the north central Gulf coast near or just west of Mobile.

The second storm track reconstruction is for the hurricane of August 1831 recorded in two fort records from Baton Rouge and Fort Pike, Louisiana (30°09′58″N, 89°44′13″W) (Fig 1b). This storm is known to have impacted Barbados on 10 August 1831 before making landfall near Last Island, Louisiana (29°03′13″N, 90°48′41″W) on 17 August 1831, as described by Chenoweth [7], who used over 80 newspapers to describe this storm. The fort in Baton Rouge reported high winds and rain with winds from the southeast on 17 August with winds switching to the southwest on 18 August. Fort Pike, located approximately 161 km east of Baton Rouge, reported rain with strong winds from the southeast on 17–18 August. These reports suggest landfall to the west of Fort Pike for a hurricane approaching from the southeast. Tree-ring records from Big Pine Key, Florida, indicate that 100% of trees were suppressed during the years 1831–1832, suggesting that a tropical cyclone passed nearby. The fort record from Key West, FL (24°32′48″N, 81°48′41″W) for 14 August 1831 remarks a “Storm” with variable winds and rain. There are reports of a hurricane passing the Tortugas on 14 August coming from Cuba and moving on to New Orleans [19]. Based on this information, we determine that the storm impacted Barbados in early August and then moved northwestward through the Caribbean into the Gulf of Mexico, making landfall to the southwest of Baton Rouge on 17 August, probably near Last Island. Despite this evidence, the lack of information while the storm was located in the open ocean still leaves the path uncertain.

The average probability track for the August 1831 tropical cyclone is determined using a weighted average of the distance grids of all track analogs [15] that passed through Barbados and then made final landfall along the central Louisiana coastline (Fig 1b). There are ten track analogs of at least tropical storm intensity in the known HURDAT2 record from 1851–2014 [5] that passed with a 300 km radius of the landfall location. The centroid of Barbados and the center of the Louisiana coastline are used as the track points [15]. Of the ten analog tracks, the average forward motion would have required eight days to traverse from Barbados to Louisiana. Given that the documental evidence suggests this storm required seven days (10–17 August) to travel from Barbados to Louisiana, this timing is highly plausible.

## Discussion

The use of historical documents as proxy evidence for tropical cyclone activity has important limitations. Documents may contain human bias and errors, such as incorrect or imprecise wind directions and mathematical errors can affect the interpretation of the document. Additionally, some of the methods used to measure meteorological variables in the 1800s are now considered archaic. For example, one study notes that the difference between true north and magnetic north can cause an error of as much as 30° in wind direction measurements [11]. In addition to wind direction, assessments of wind speed (e.g., Beaufort scale) have changed with time, so wind speeds must be converted to modern scales [20]. Although the disadvantages of using historical documents must be considered, many advantages do exist. The accuracy of such records is likely enhanced because the Army fort surgeon, who had an advanced education and scientific training, typically kept the fort meteorological records. These records include a standard form with instructions and training for observers. The frequent and consistent nature of these fort records, as well as the details in a number of records, offer a consistency check that reduces the potential for human or instrumental bias.

The storm track reconstruction relies largely on the climatology of modern hurricane tracks, but not all unique characteristics are lost. The incorporation of known landfall points provides insight into the potential damage path and temporal variation in track patterns. Such a reconstruction can improve understanding of long-term variability in tropical cyclone frequency. The method could be improved by considering the posterior probability distribution that the historical hurricane track reached within a specified distance of any location in the basin. This Bayesian type approach could provide a statistical framework for understanding tropical cyclone behavior in other historical hurricane chronologies [15].

## Conclusions

Historical documents prove to be useful for detecting previously unknown or poorly documented tropical cyclones. Our review of fort records, plantation records, and storm reports in the NOAA EV2 database reveals 25 tropical cyclones and three undocumented possible pre-1851 tropical cyclones. We find that the EV2 database is a useful tool in detecting past storms along the US Gulf Coast; however, the qualitative nature of these records invites caution in interpretation of results. Coupling historical documents with other paleoclimate proxies can increase our understanding of tropical cyclones. We reconstruct the likely storm track for the 1831 tropical cyclone based on information taken directly from the historical documents and a tree-ring reconstruction along with average track analogs of known storms from 1851–2014. The addition of historical documents to enhance the HURDAT2 database can help reduce the observational biases in published tropical cyclone frequencies for longer intervals of time, which is critical for intervals before anthropogenic warming and during the colder Little Ice Age.

## Supporting Information

S1 Table Metadata of reports used from EV2 database. (PDF)

S2 Table Ten analog tropical cyclone events (at least tropical storm strength) used to create the average probability track provided in Fig 1(b). The storm identification number (SID) is included for reference to the HURDAT archive, with the name and year of the event. Storms listed as "unnamed" occurred prior to the naming protocol standardized in 1950.
(PDF)
