# Supplementary material for: Using Proxy Records to Document Gulf of Mexico Tropical Cyclones from 1820-1915
Source: PLoS One. 2016 Nov 29;11(11):e0167482. doi: 10.1371/journal.pone.0167482 (PMC5127585; doi:10.1371/journal.pone.0167482)
Supplement: S1 Table — (PDF) [file pone.0167482.s001.pdf]

Type	Year	Location	Document ID	Batch Number	Coordinates
Storm Report	1915	Louisiana	100001603	AC62059C	
Storm Report	1909	Louisiana	100006208	AC62064B	
Storm Report	1908	Louisiana/Texas	100006935	AC62064A	
Storm Report	1902	Texas	100004753	AC62072C	
Storm Report	1898	Florida	100005531	AC62071B	
Storm Report	1896	Florida	100007070	AC62071A	
Storm Report	1894	Florida	100006679	AC62070B	
Fort	1890	Baton Rouge, Louisiana	93082	AE04487	30º27′29”N, 91º08′25”W
Storm Report	1888	Florida	100003379	AC62070AB	
Plantation	1888	Ashton, Louisiana	90695	AE04487	29º50′09”N, 91º36′29”W
Fort	1887	Grand Coteau, Louisiana	85522	AE04486	30º25′11”N, 92º02′47”W
Fort	1880	Brownsville, Texas	411842	AE08576	25º54′06”N, 97º29′50”W
Fort	1879	Okaloosa, Louisiana	94707	AE04483	32º22′48”N, 92º19′40”W
Fort	1877	Baton Rouge, Louisiana	93018	AE04487	30º27′29”N, 91º08′25”W
Fort	1875	Brownsville, Texas	554504	AE08570	25º54′06”N, 97º29′50”W
Fort	1870	Fort Jefferson, Florida	596994	AE03224	24º37′40″N, 82º52′22″W
Fort	1865	Galveston, Texas	650	AE06178	29º18′04″N, 94º47′51″W
Fort	1863	Key West, Florida	257102	AE03220	24º32′48″N, 81º48′41″W
Fort	1859	Tickfaw, Louisiana	94728	AE04482	30º34′38”N, 90º28′59”W
Fort	1856	Baton Rouge, Louisiana	91668	AE04487	30º27′29”N, 91º08′25”W
Fort	1852	Eutaw, Alabama	12480	AE03152	32º50′26″N, 87º53′15″W
Fort	1852	Pascagoula, Mississippi	336762	AE04572	30º21′56″N, 88º33′22″W
Fort	1837	Fort Jesup, Louisiana	84176	AE04486	31º36′41″N, 93º24′03″W
Fort	1831	Baton Rouge, Louisiana	91018	AE04487	30º27′29”N, 91º08′25”W
Fort	1831	Fort Pike, Louisiana	84541	AE04486	30º09′58”N, 89º44′13”W
Fort	1831	Key West, Florida	260354	AE03221	24º32′48″N, 81º48′41″W
Fort	1828	Fort Pike, Louisiana	84476	AE04486	30º09′58”N, 89º44′13”W
Fort	1825	Petite Coquille, Louisiana	84419	AE04486	30º09′58”N, 89º44′13”W
Fort	1822	Fort St. Philip, Louisiana	84912	AE04486	29º21′47″N, 89º27′57″W
Fort	1822	Dauphin Island, Mississippi	12197	AE03152	30º15′15″N, 88º06′44”W
Fort	1822	Camp Hope, Florida	596878	AE03224	30°23'52"N, 87°14'27"W
Fort	1820	Bay St. Louis, Mississippi	135643	AE04573	30º18′31″N, 89º19′48″W
