# Supplementary material for: Using Proxy Records to Document Gulf of Mexico Tropical Cyclones from 1820-1915
Source: PLoS One. 2016 Nov 29;11(11):e0167482. doi: 10.1371/journal.pone.0167482 (PMC5127585; doi:10.1371/journal.pone.0167482)
Supplement: S2 Table — The storm identification number (SID) is included for reference to the HURDAT archive, with the name and year of the event. Storms listed as "unnamed" occurred prior to the naming protocol standardized in 1950. (PDF) [file pone.0167482.s002.pdf]

**Analog Number**	**SID**	**Name**	**Year**
1	164	Unnamed	1875
2	286	Unnamed	1889
3	482	Unnamed	1915
4	486	Unnamed	1915
5	505	Unnamed	1917
6	506	Unnamed	1918
7	690	Unnamed	1938
8	1572	Lili	2002
9	1577	Claudette	2003
10	1603	Ivan	2004
